# Antifungal and Antiproliferative Protein from *Cicer arietinum*: A Bioactive Compound against Emerging Pathogens

**DOI:** 10.1155/2014/387203

**Published:** 2014-05-14

**Authors:** Suresh Kumar, Vaishali Kapoor, Kamaldeep Gill, Kusum Singh, Immaculata Xess, Satya N. Das, Sharmistha Dey

**Affiliations:** ^1^Department of Biophysics, All India Institute of Medical Sciences, New Delhi 110029, India; ^2^Department of Biotechnology, All India Institute of Medical Sciences, New Delhi 110029, India; ^3^Department of Microbiology, All India Institute of Medical Sciences, New Delhi 110029, India

## Abstract

The emergence of epidemic fungal pathogenic resistance to current antifungal drugs has increased the interest in developing alternative antibiotics from natural sources. *Cicer arietinum* is well known for its medicinal properties. The aim of this work was to isolate antimicrobial proteins from *Cicer arietinum*. An antifungal protein, C-25, was isolated from *Cicer arietinum* and purified by gel filtration. C-25 protein was tested using agar diffusion method against human pathogenic fungi of ATCC strains and against clinical isolates of *Candida krusei*, *Candida tropicalis*, and *Candida parapsilosis*, and MIC values determined were varied from 1.56 to 12.5 **μ**g/mL. The SEM study demonstrated that C-25 induces the bleb-like surface changes, irregular cell surface, and cell wall disruption of the fungi at different time intervals. Cytotoxic activity was studied on oral cancer cells and normal cells. It also inhibits the growth of fungal strains which are resistant to fluconazole. It reduced the cell proliferation of human oral carcinoma cells at the concentration of 37.5 **μ**g/mL (IC_50_) and no toxic effect was found on normal human peripheral blood mononuclear cells even at higher concentration of 600 **μ**g/mL. It can be concluded that C-25 can be considered as an effective antimycotic as well as *antiproliferative* agent against human oral cancer cells.

## 1. Introduction

The major healthcare problem is the antibiotic resistance which arises the lack of effective therapeutics for microbial infection. During the past few years a wide spectrum of plant antimicrobial proteins has been identified and has enhanced the activity in low duration to prevent the development of resistant by microbes.

There are several classes of proteins having antimicrobial properties which include thionins, lipid transfer proteins, plant defensins, chitinases, glucanases, 2S albumins, ribosome inactivating proteins, and lectin [1, 2]. Lectins are proteins or glycoproteins of a ubiquitous distribution in nature, which have at least one carbohydrate or derivative binding site without catalytic function or immunological characteristics. They have the unique ability to recognize and bind reversibly to specific carbohydrate ligands without any chemical modification which distinguishes lectins from other carbohydrate binding proteins and enzymes and makes them invaluable tools in biomedical and glycoconjugate research. In plant, lectin plays an important role in the defence against harmful fungi, insects, and bacteria. Several lectins have been found to possess anticancer properties in human case studies, where they are used as therapeutic agents binding to the cancer cell membrane or their receptors causing cytotoxicity, apoptosis, and inhibition of tumor growth [3, 4].

*Cicer arietinum* (chickpea) is a legume and belongs to the Fabaceae family. It contains 75% fibres and low fat protein. It has been reported that the use of* Cicer arietinum* helps in diabetes and cardiovascular diseases and in some cancers. Some lectins having hemagglutination activity were isolated earlier from* Cicer arietinum* [5]. This study focused on isolation and characterization of a lectin protein possessing medicinal properties from the seeds of* Cicer arietinum*.

## 2. Methods

### 2.1. Ethics

The Ethics Committee of All India Institute of Medical Sciences (AIIMS), New Delhi, India, approved the study protocol (IEC/NP-374/2013) and informed consent was obtained.

### 2.2. Isolation and Purification of Protein from* Cicer arietinum*

*Cicer arietinum* seeds were soaked, homogenized in 10 mM Tris-Cl buffer (pH7.2), and centrifuged at 13,000 ×g for 30 min. at 4°C. The resulting crude extract was treated with ammonium sulphate with 30% saturation under cold condition and the precipitant was centrifuged at 13,000 ×g for 30 min. at 4°C. The salt was removed from the resultant supernatant by dialysis membrane (10 kDa) in the same buffer.

The dialysed sample was loaded onto Sephadex G-100 gel filtration column preequilibrated with 10 mM Tris-HCl (pH7.2) and 150 mM NaCl. The proteins were eluted using the same buffer and simultaneously monitored at 280 nm. Each fraction was tested for antimicrobial activity. One fraction showed inhibition activity against fungi and it was characterized further.

### 2.3. Characterization of the Purified Protein 

#### 2.3.1. Molecular Mass Determination

The concentration of proteins was estimated by BCA Protein Assay Kit (Thermo Scientific, Rockford, USA) using Bovine serum albumin as a standard. The 12% SDS-PAGE of the protein was carried out using Laemmli system of buffers [6] in the presence and absence of 2-mercaptoethanol. The electrophoretic mobility of the protein and protein marker were compared to determine the molecular weight of the protein.

#### 2.3.2. N-Terminal Amino Acid Sequence Analysis

The N-terminal sequence analysis of the C-25 protein was done by Edman degradation on a Procise Protein Sequencer (Applied Biosystems). The database was searched for other antifungal proteins with similar sequences using BLAST (http://www.ncbi.nlm.nih.gov/BLAST).

#### 2.3.3. Hemagglutination Activity and Sugar Inhibition Assays

Hemagglutination studies of the purified protein were carried out using human erythrocytes in a 96-well microtiter plate. 50 *μ*L of purified protein solution (0.8 mg/mL) was placed in the first well and twofold serially diluted into the successive wells with phosphate buffered saline, pH 7.4. Then, 50 *μ*L of 4% human erythrocyte suspension was added to all the wells. Hemagglutination was visualized in the plate after 1 h of incubation at 37°C.

Hemagglutination inhibition assays [7] with the purified protein were performed by placing 50 *μ*L of different sugar solutions (40 mM) including inulin, D-mannose, D-glucose, D-ribose, N-acetyl-D-galactosamine, and melibiose in respective wells of the plate and serially twofold diluted. Then, 50 *μ*L of the purified protein (0.8 mg/mL) was added to each well and incubated for 30 min. at 37°C. Later, 50 *μ*L of 4% erythrocyte suspension was added and the plate was incubated for 1 h at 37°C. Hemagglutination inhibition titre was scored visually.

#### 2.3.4. Detection of Antifungal Activity

Antifungal activity of the purified protein was tested using agar diffusion method against human pathogenic fungi such as* Candida parapsilosis* ATCC22019,* Candida krusei* ATCC6258, and* Candida tropicalis* ATCC13803. All* Candida* species were grown overnight on Sabouraud's dextrose agar plates. Each colony was inoculated in 5 mL of 0.9% (w/v) normal saline to make inoculum suspension adjusted with 0.5 Mc Farland standard solutions and the cell suspension was spread by sterile cotton swab over the Mueller Hinton agar (MHA) plates under aseptic conditions. The wells were bored with a borer and 0.1 mL of purified protein (200 *μ*g/mL) was added to respective wells. Fluconazole disc (25 mcg) was used as positive control. The plates were incubated at 35°C for 24 h and the zone of inhibition was observed.

#### 2.3.5. Determination of Minimum Inhibitory Concentration (MIC)

The MIC was performed on* Candida parapsilosis* ATCC22019,* Candida krusei* ATCC6258,* Candida tropicalis* ATCC13803, and clinical isolates of the same strains from 45 patients with* Candida* infection. The patient samples were taken from Department of Microbiology, AIIMS. The MIC was determined according to the CLSI (Clinical and Laboratory Standards Institute) guidelines [8]. Pure colonies of* Candida* species were suspended in 5 mL of sterilized saline (0.9% w/v) to a concentration of 5 × 10^6^ CFU/mL as matched with 0.5 McFarland Standard solutions. 100 *μ*L of purified protein (200 *μ*g/mL) solution was added and twofold serially diluted using RPMI-1640 media. 100 *μ*L of each final inoculum suspension (2.5 × 10^3^ CFU/mL) was added to the respective wells and the plates were incubated at 35°C for 24 h. The fungal strains* C. krusei* and* C. parapsilosis* without treatment and uninoculated RPMI-1640 media were used as growth and media control, respectively. The MIC was calculated as the lowest concentration at which cell growth was inhibited. Fluconazole drug was used as a positive control. The experiment was performed in triplicate.

#### 2.3.6. Scanning Electron Microscopic (SEM) Studies

The inoculated* Candida krusei* ATCC6258 was incubated in MHB (Mueller Hilton broth) media at 35°C overnight, which was further incubated for another 2 h at 35°C in fresh media for exponential growth phase. After washing with PBS, cells were suspended in 10 mM phosphate buffer (pH 7.4) at a final concentration of 1 × 10^6^ CFU/mL. The protein at concentrations of 12.5 *μ*g/mL (2xMIC) was added to the cell suspension and was incubated at different time intervals at 35°C. High concentration of test sample is chosen in order to achieve killing of a high number of yeast cells [9]. The cells were prepared for SEM study by treatment with 1% osmium tetroxide for 1 h at 4°C [10]. The cells were visualized under Electron Microscope (LEO, Cambridge, UK). Images were digitally acquired by using a CCD camera attached to the microscope.

#### 2.3.7. Determination of Cytotoxicity

Cytotoxic effect of protein was analyzed using oral carcinoma cell line (KB cells) using MTT dye reduction assay [11]. Briefly, 5 × 10^3^ cells/100 *μ*L media (EMEM) were seeded in 96-well plates 24 h before the experiment. The cells were then incubated with different concentrations (9–600 *μ*g/mL in EMEM) of protein for 48 h. 10 *μ*L of MTT solution (10 mg/mL in PBS) was then added to each well and plates were further incubated for 3 h at 37°C. The formazan crystals formed were dissolved by adding 100 *μ*L of DMSO. The cells were treated with 1% Tris-HCl (same concentration as used for the solvent of C-25) which was then subtracted from all the cytotoxic values. Absorbance was measured by a microplate reader at 570 nm and the reference filter 650 nm was used. The data obtained were presented as percentage of cell survival in the best-fit (linear) dose response curves. The IC_50_ value at 95% confidence interval was calculated. Each concentration was used in triplicate.

To examine the cytotoxicity effect of this protein on PBMCs, peripheral blood mononuclear cells (PBMCs) were isolated by density gradient centrifugation using Histopaque-1077 (Sigma-Aldrich, USA) as per the standard protocol [12] from healthy human blood and collected in heparinized tubes and diluted 1 : 2 with fresh sterile phosphate-buffered saline (PBS). The proliferation analysis of C-25 was performed by MTT assay as described above.

#### 2.3.8. Kinetic Analysis with p38*α* MAP Kinase

The kinetic analysis of protein was done with p38*α* MAP kinase as this signaling molecule was found to be overexpressed in oral cancer [13]. Hence, binding study of protein was performed with recombinant p38*α* MAP kinase (mitogen activated protein kinase) using both ELISA and BIAcore to ensure the anticancer activity.

*(1) By ELISA.* The assay was performed in 96-well microtitre plate coated with ATF-2 protein at 37°C. 12 *μ*g of p38*α* protein was incubated with six different concentrations of protein (1, 2.5, 5, 10, 15, 20, and 25 *μ*M) for 1.5 h. The kinase mixture (purified p38*α* incubated with C-25 protein, 50 mM Tris, pH 7.5, 10 mM MgCl_2_, 10 mM *β*-glycerophosphate, 100 *μ*g/mL BSA, 1 mM DTT, 0.1 mM  Na_3_VO_4_, and 100 *μ*M ATP) was added and incubated for 1 h at 37°C. After washing, the plates were incubated with anti-phospho ATF-2 antibody (1 : 400) (Biovision) for 1 h at 37°C and subsequently with alkaline phosphates conjugated goat anti-rabbit IgG (1 : 4000) (Chemicon) for 1 h at 37°C. Finally, the chromogenic substrate solution 4-nitrophenyl phosphate (4-NPP) in 0.1 M Tris-HCl, pH 8.1, and 0.01% MgCl_2_ (Cayman Chemical Company, USA) was added for 1.5 h at 37°C and the formation of nitrophenolate was measured at 405 nm which analyzed the extent of phosphorylation of ATF-2. The assay was performed in triplicate for each concentration and mean ± SD values were used to calculate the IC_50_ value.

*(2) By Surface Plasmon Resonance (SPR).* The His-tagged recombinant p38*α* protein was immobilized on the NTA sensor chip via Ni^2+^/NTA chelation at 25°C in BIAcore-2000 (GE Healthcare, Sweden). The surface was first activated with Ni^2+^ forming a chelating complex with NTA which further binds with His-tag of recombinant protein. 2 mM NiCl_2_ solution was passed at a flow rate of 5 *μ*L/min. One flow cell was used as a reference cell and, on the other, 20 *μ*L of His-tagged p38*α* (9 mg/mL) was injected at a flow rate of 5 *μ*L/min. for immobilization. The binding parameters of the C-25 were measured by injecting three different concentrations (4.6 × 10^−6^ M, 9.2 × 10^−6^ M, and 13.8 × 10^−6^ M) over the immobilized protein. BIAevaluation 3.0 software was used to determine the dissociation constant (KD) of the inhibitory protein.

## 3. Results

### 3.1. Purification and Molecular Characterization

**Figure 1:**
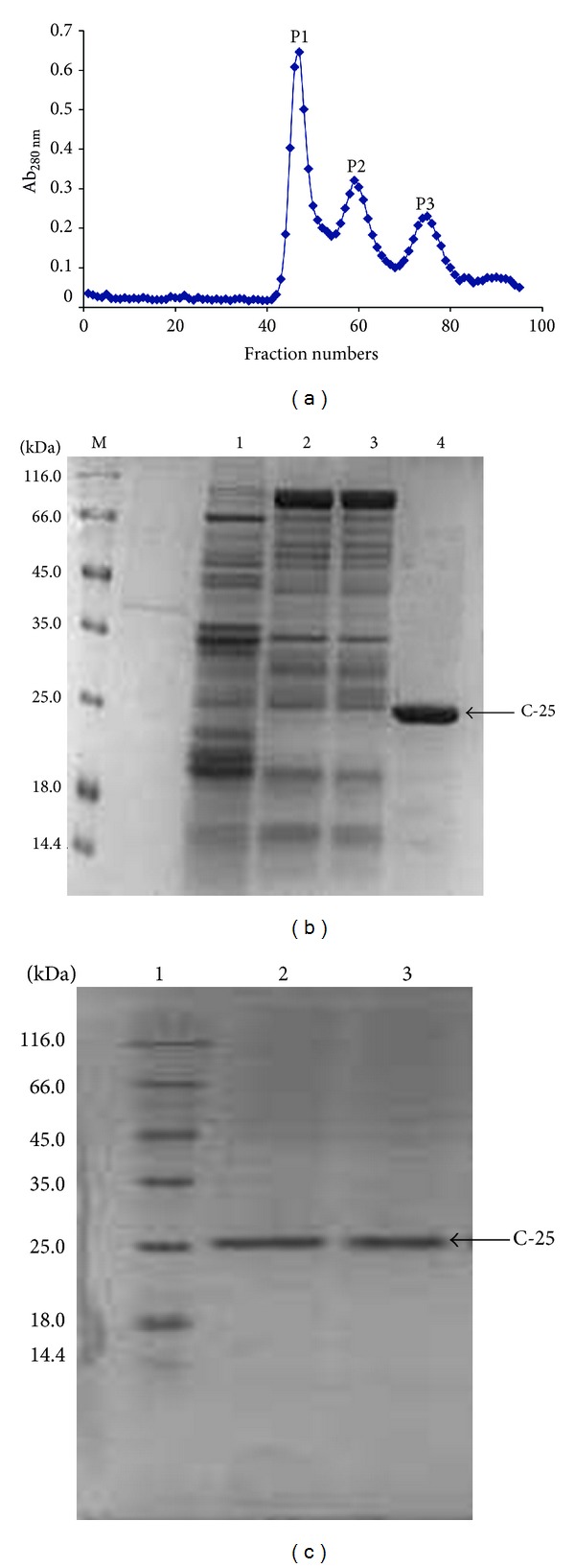
(a) Elution profile of* C. arietinum *protein crude extract from gel filtration on Sephadex G-100 column showing three peaks P1, P2, and P3. SDS-PAGE of protein fractions from gel filtration: (b) from left to right: Lane M is molecular mass marker, Lane 1 is eluent of P1, Lanes 2 and 3 are P2, and Lane 4 is P3 (C-25). (c) From left to right: Lane 1 is molecular mass marker, Lane 2 is P3 in the absence of mercaptoethanol under nonreducing conditions, Lane 3 is P3 in the presence of mercaptoethanol under reducing conditions.

Crude protein extract from* Cicer arietinum* was subjected to ammonium sulphate precipitation to remove unwanted proteins. Three peaks were obtained after gel filtration with Sephadex G-100 column (Figure 1(a)). In SDS-PAGE of these fractions, the third peak showed a single band corresponding to molecular mass of 25 kDa named as C-25. The antifungal activity was found in peak 3 fractions (Figure 1(b)). Both Lane 2 and Lane 3 showed a single band of C-25 in the presence and absence of mercaptoethanol, respectively, which revealed the protein to be a monomer (Figure 1(c)).

### 3.2. N-Terminal Amino Acid Sequence Analysis

N-terminal amino acid sequence of the purified C-25 from* Cicer arietinum* is shown in Table 1 and it was compared with other antifungal proteins using Blast from NCBI website. This protein exhibited 100% sequence similarity of 10 amino acid residues with sequence of lectin from other plant sources.

### 3.3. Hemagglutinating Activity and Inhibition Assay

C-25 protein from* Cicer arietinum* readily agglutinated human erythrocytes showing the hemagglutination activity. Hemagglutination-inhibition assay was performed with C-25 to investigate its sugar specificity. The results showed that agglutination activity of C-25 was inhibited strongly by N-acetyl-D-galactosamine and not by any other sugar moieties, indicating that the acetamido moiety of this sugar might have interacted with C-25. Agglutination activity of C-25 was inhibited by 20 mM of N-acetyl-D-galactosamine (S1) indicating that C-25 specifically binds with N-acetyl-D-galactosamine.

### 3.4. Assay of Antifungal Activity

**Figure 2:**
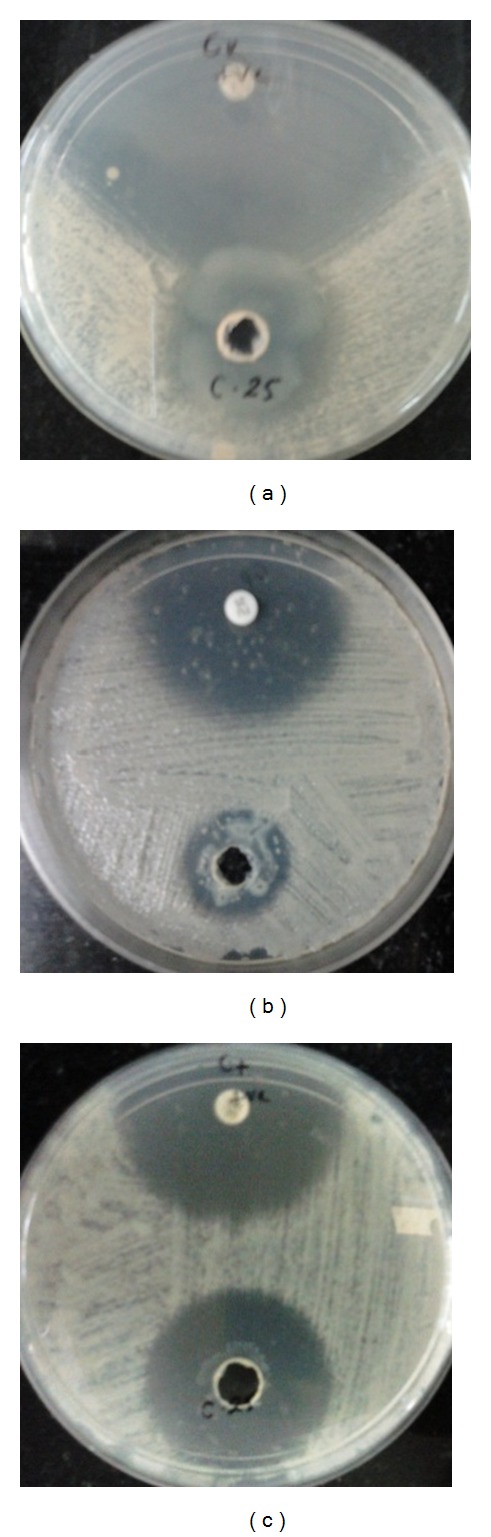
Antifungal assay of the C-25 protein showing zone of inhibition against (a)* Candida krusei*, (b)* C. parapsilosis*, and (c)* C. tropicalis*. Fluconazole disc was taken as positive control.

The pure C-25 obtained from gel filtration was tested for antifungal activity against* C. parapsilosis*,* C. krusei*, and* C. tropicalis *by agar well diffusion method using fluconazole drug as a positive control. The zone of inhibition around the test sample was found in all the above mentioned* Candida *spp. (Figures 2(a), 2(b), and 2(c)). The MIC values of a C-25 against the above mentioned fungi and clinical isolates of* Candida* species from 45 patients were found to be varied from 1.56 to 12.5 *μ*g/mL after 24 h incubation period. Among 45 clinical isolates strains, the MIC of ≤8 *μ*g/mL are susceptible, 16 to 32 *μ*g/mL are susceptible-dose dependent (SDD), and ≥64 *μ*g/mL are resistant to fluconazole as per CLSI document M27-A3, although C-25 was showing fungicidal activity on these strains (Table 2).

### 3.5. SEM Studies

**Figure 3:**
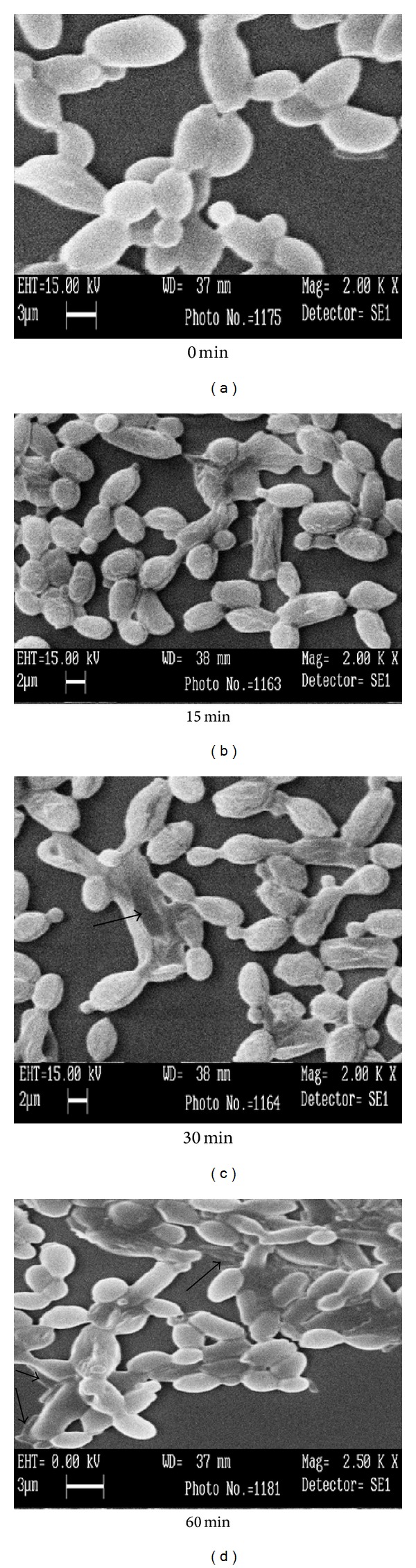
SEM study showing the cell wall disruption of* Candida krusei* treated with (a) 10 mM PBS buffer (control) and (b), (c), and (d) 2xMIC value (12.5 *μ*g/mL) of C-25 protein at different time scales. The arrows indicate the cell wall disruption and cytoplasmic leakage.

To understand the mechanism of action of C-25 on the cell wall of fungi, SEM studies were performed with cells of* C. krusei* at different times of incubation with 12.5 *μ*g/mL of C-25 and the changes in the morphology of cell wall of the* C. krusei* were examined.  Figure 3(a) showed the morphology of the untreated cells (control). The effect of 12.5 *μ*g/mL concentrations of C-25 showed different consequences on the cell wall.  Figure 3(b) showed the bleb-like surface changes and cell shrinkage at 15 min, and Figures 3(c) and 3(d) showed irregular cell surface, cell wall disruption, and cytoplasmic leakage at different times, 30 and 60 min., respectively.

### 3.6. Cytotoxicity

**Figure 4:**
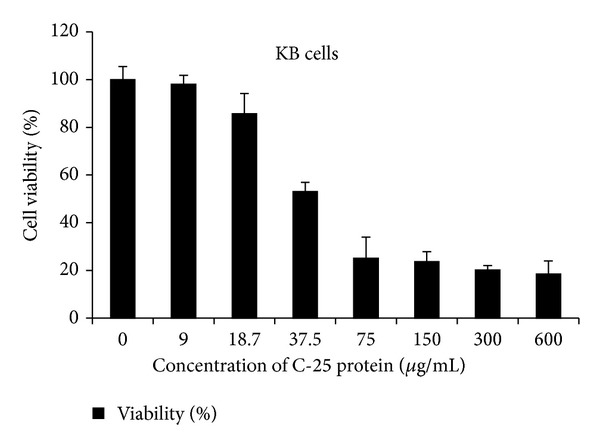
Cell viability of cancer cells (KB cell line) treated with different concentrations of C-25 protein.

The cytotoxicity of the C-25 against KB cell line was investigated using MTT assay. 50% of KB cell survival was reduced by treating with 37.5 *μ*g/mL (IC_50_) of C-25. At 75 *μ*g/mL, it significantly inhibited the survival of KB cells in 48 h incubation period (Figure 4).

**Figure 5:**
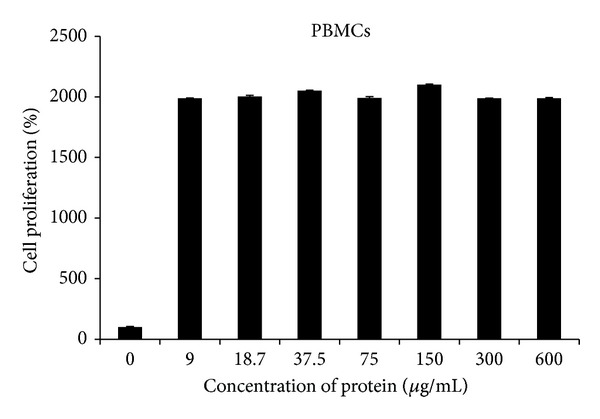
Cell proliferation of normal PBMCs at different concentrations of C-25 protein.

In the case of normal mammalian cells (PBMCs), no toxic effect of C-25 lectin was found even at higher concentration of 600 *μ*g/mL but it enhanced the normal cell proliferation (Figure 5). Hence, it indicates that the C-25 inhibits the proliferation of cancer cells selectively.

### 3.7. Kinetic Analysis of C-25 with p38*α* MAP Kinase

p38*α* is a cell signaling molecule and is reported to be overexpressed in oral cancer [13]. Hence, binding study of C-25 was performed with recombinant p38*α* MAP kinase (mitogen activated protein kinase) using both ELISA and BIAcore to ensure the antiproliferative activity.

#### 3.7.1. By ELISA

**Figure 6:**
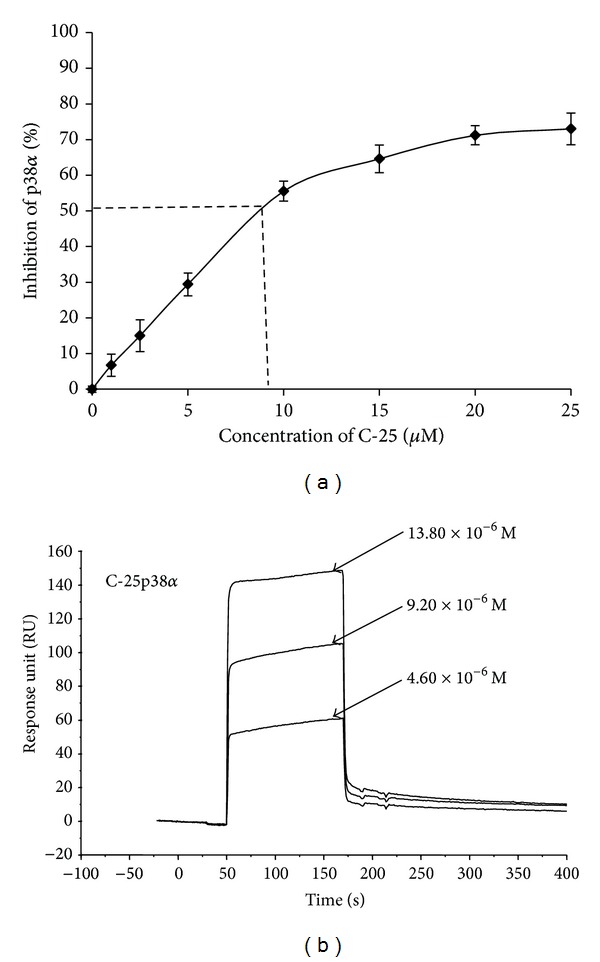
Inhibition assay: (a) % inhibition of p38*α* with increasing concentration of C-25; (b) Sensorgram showing the binding interaction of p38*α* with increasing concentrations of C-25.

The pure p38*α* was incubated with C-25 and the phosphorylation activity of p38*α* was tested in the presence of ATP. It inhibited p38*α* by competing with ATP. Thus, it prevented the phosphorylation of the activated transcription factor-2 (ATF-2). The IC_50_ value of C-25 was found to be 7.9 *μ*M against the pure p38*α* protein (Figure 6(a)).

#### 3.7.2. By SPR Technology

The specific bindings of C-25 were determined in the form of binding capacity on to immobilized p38*α* protein. The change in RU (resonance unit) with different concentrations denoted the change in bound mass on the sensor chip with time giving the KD value of C-25, 2.69 × 10^−7^ M. The sensorgram in Figure 6(b) shows the binding of varying concentrations of C-25 over p38*α*.

Hence, by ELISA and SPR it can be revealed that C-25 can inhibit the activity of p38*α*.

## 4. Discussion

*Cicer arietinum* has been used in many traditional medical purposes. C-25 protein isolated from* Cicer arietinum* exhibited strong antifungal activities against human pathogens:* Candida krusei*,* Candida tropicalis*, and* Candida parapsilosis* of MIC values 1.56–12.5 *μ*g/L. It also inhibits the growth of fungal strains which are resistant and susceptible-dose dependent to fluconazole. The MIC of C-25 on fungal growth was comparable to the antifungal lectins of other leguminous plants. Though the exact mode of action of lectin on fungal growth is not clearly known it was previously observed by SEM that lectin disrupted the cell wall and resulted in leakage of cytoplasm [14]. In the present investigation, C-25 also acts primarily on the cell wall of* Candida* species, by disrupting the cell wall and distorting the cellular morphologies.

Lectins are widely used in agriculture as antimicrobials and pesticides. Some lectins have been isolated from plants having antifungal properties in plant pathogens [15–22]. The present study reveals the isolation of lectin (C-25) of molecular weight 25 kDa from* Cicer arietinum*. The C-25 was found to be monomer as the molecular mass obtained by SDS-PAGE analysis was the same in both reducing and nonreducing conditions. N-terminal sequence of the C-25 protein had some amino acids sequence similarity with the previously isolated lectin from other plant sources having a different molecular weight. The database search using BLAST indicated that the sequence showed 100% homology with lectins of* Pisum sativum*,* Lathyrus sativus*, and* Cicer arietinum*. The characteristic properties of lectin isolated previously from* Cicer arietinum* (PDB 3S18) are not reported. The present study isolated lectin C-25 from chickpea (*Cicer arietinum*) and reported the biological properties. Many sugar binding lectins from seeds of leguminous plants are well characterised and offer many biological functions. The hemagglutination activity of C-25 was inhibited by N-acetyl-D-galactosamine and showed to be N-acetyl-D-galactosamine-specific protein.

It is well recognized that lectins exhibit an anticancer activity. The intensive cancer research is going on the basis of different cell surface sugar moieties of cancerous cells [23]. The different mode of cytotoxic effect was observed by different lectin. Lectin isolated from different sources differentially inhibited the type of cancer cell proliferation like leukemia L1210 cells [24], HeLa and FemX cells [25], breast cancer MCF7 cells and hepatoma HepG2 cells [26], hepatoma (HepG2) cells [27, 28], and KB cell line. Earlier studies have reported the inhibitory effect of ethanol/acetone extract from* Cicer arietinum* on the proliferation of Caco-2 cells [29] as well as the antiproliferative effect of* Cicer arietinum* PIC on breast and prostate cancer cell lines [30]. In the present study, MTT assay demonstrated a significant cell death of oral cancer cell line (KB cell line) treated with C-25. The inhibition of KB cell line viability with C-25 was concentration dependent. But even at high doses it is nontoxic to normal mammalian PBMCs; rather it induces proliferation of normal cells which is the characteristic of many plant lectins [31].

This lectin also inhibits the p38*α* MAP kinase in presence of substrate (ATP) and showed binding affinity with p38*α*. The p38*α* plays a central role in the production of inflammatory cytokines IL-1*β*, TNF-*α*, and IL-6. The overproduction of these cytokines causes tumor growth. There is an evidence of overexpression of p38*α* in oral cancer patients and its declination after treatment [13]. Hence, it may be assumed that C-25 inhibits the oral cancer cell lines (KB cells) growth by targeting p38*α* MAP kinase.

It can be concluded that a lectin C-25 isolated from* Cicer arietinum* possessed carbohydrate specificity and antifungal and antiproliferative activity. Hence, C-25 only after* in vivo* studies can be considered to be an effective bioactive compound.

## Supplementary Material

Table S1-Hemagglutination inhibition assay on C-25 with various sugar moieties

## Figures and Tables

**Table 1:** A comparison between N-terminal amino acids sequence of C-25 (TKTGYINAAF) and sequences of other proteins.

Protein	Sequence	Accession number	% identity
C-25 protein	TKTGYINAAF	AGN33419	100
Seed albumin 2 *(Pisum sativum) *	TKTGYINAAF	CAH55839.1	100
Albumin 2 *(Pisum sativum) *	TKTGYINAAF	P08688.1	100
Crystal structure of Ls24 (*Lathyrus sativus) *	TKPGYINAAF	Pdb:3LP9A	90
Crystal structure of albumin *(Cicer arietinum) *	TKTGYINAAF	3S18	100

**Table 2:** MIC assay: ATCC and clinical isolates of *Candida* species from 45 patients treated with C-25 protein and fluconazole drug (control).

ATCC number/ patient ID	Organism	Source	MIC (*μ*g/mL) of fluconazole	MIC (*μ*g/mL) of C-25
22019 (QC)	*C. parapsilosis *	ATCC	1	6.25
6258 (QC)	*C. krusei *	ATCC	16	6.25
13803 (reference)	*C. tropicalis *	ATCC	1	6.25
AID 19	*C. tropicalis *	Blood	2	6.25
AID 20	*C. tropicalis *	Blood	2	6.25
AID 21	*C. tropicalis *	Blood	2	6.25
AID 37	*C. parapsilosis *	Blood	1	6.25
AID 45	*C. tropicalis *	Blood	1	6.25
AID 47	*C. tropicalis *	Blood	1	6.25
2549	*C. tropicalis *	Blood	0.5	1.56
4347	*C. tropicalis *	Blood	1	3.125
7004	*C. tropicalis *	Blood	0.5	1.56
9097	*C. parapsilosis *	Urine	1	3.125
9409	*C. parapsilosis *	Urine	1	3.125
8995	*C. parapsilosis *	Urine	4	6.25
8399	*C. tropicalis *	Urine	4	6.25
8509	*C. tropicalis *	Urine	2	6.25
8110	*C. tropicalis *	Urine	2	6.25
9853	*C. tropicalis *	Urine	1	6.25
9697	*C. tropicalis *	Urine	1	3.125
9183	*C. tropicalis *	Urine	1	3.125
9814	*C. tropicalis *	Urine	1	3.125
9239	*C. tropicalis *	Urine	2	6.25
9762	*C. tropicalis *	Urine	1	3.125
