# Supplementary material for: Antifungal and Antiproliferative Protein from Cicer arietinum: A Bioactive Compound against Emerging Pathogens
Source: Biomed Res Int. 2014 May 14;2014:387203. doi: 10.1155/2014/387203 (PMC4053179; doi:10.1155/2014/387203)
Supplement: Supplementary file 1 — Table S1-Hemagglutination inhibition assay on C-25 with various sugar moieties [file 387203.f1.doc]

Table 2. Hemagglutination inhibition assay on C-25 with various sugar moieties.

| Concentration of sugar (mM) |
| --- |

Test sugars 40 20 10 5 2.5 1.25 0.625 0.312 0.156 0.07 PBS

|  |
| --- |

D-glucose − − − − − − − − − − −

D-mannose − − − − − − − − − − −

N-acetyl galactosamine + + − − − − − − − − −

D-ribose − − − − − − − − − − −

Inulin − − − − − − − − − − −

Melibiose − − − − − − − − − − −

| +, hemagglutination inhibition; −, no hemagglutination inhibition; PBS, phosphate buffer saline. |
| --- |
